# Spermatogonial Stem Cell-Based Therapies: Taking Preclinical Research to the Next Level

**DOI:** 10.3389/fendo.2022.850219

**Published:** 2022-04-04

**Authors:** Iris Sanou, Jillis van Maaren, Jitske Eliveld, Qijing Lei, Andreas Meißner, Annemieke A. de Melker, Geert Hamer, Ans M. M. van Pelt, Callista L. Mulder

**Affiliations:** ^1^ Reproductive Biology Laboratory, Center for Reproductive Medicine, Amsterdam University Medical Center (UMC), Amsterdam Reproduction and Development Research Institute, University of Amsterdam, Amsterdam, Netherlands; ^2^ Department of Urology, Center for Reproductive Medicine, Amsterdam University Medical Center (UMC), Amsterdam Reproduction and Development Research Institute, University of Amsterdam, Amsterdam, Netherlands

**Keywords:** spermatogonial stem cells, fertility preservation, fertility restoration, childhood cancer, preclinical research, spermatogonial stem cell transplantation, testicular grafting, *in vitro* spermatogenesis

## Abstract

Fertility preservation *via* biobanking of testicular tissue retrieved from testicular biopsies is now generally recommended for boys who need to undergo gonadotoxic treatment prior to the onset of puberty, as a source of spermatogonial stem cells (SSCs). SSCs have the potential of forming spermatids and may be used for therapeutic fertility approaches later in life. Although in the past 30 years many milestones have been reached to work towards SSC-based fertility restoration therapies, including transplantation of SSCs, grafting of testicular tissue and various *in vitro* and *ex vivo* spermatogenesis approaches, unfortunately, all these fertility therapies are still in a preclinical phase and not yet available for patients who have become infertile because of their treatment during childhood. Therefore, it is now time to take the preclinical research towards SSC-based therapy to the next level to resolve major issues that impede clinical implementation. This review gives an outline of the state of the art of the effectiveness and safety of fertility preservation and SSC-based therapies and addresses the hurdles that need to be taken for optimal progression towards actual clinical implementation of safe and effective SSC-based fertility treatments in the near future.

## Introduction

In recent decades, spermatogonial stem cell (SSC)-based therapeutic fertility approaches have become an important topic of investigation to overcome gonadotoxic treatment-induced infertility. The increasing survival rates over time of young cancer patients have highlighted the impact of gonadotoxic cancer treatments and the consequences for their fertility later in life. Currently, there is a survival rate of 80% among pediatric cancer patients in Europe (1), leading to a large population of childhood cancer survivors who are at risk of cancer treatment related infertility, for whom these SSC-based therapeutic approaches may be an opportunity to father genetically related children.

**Figure 1:**
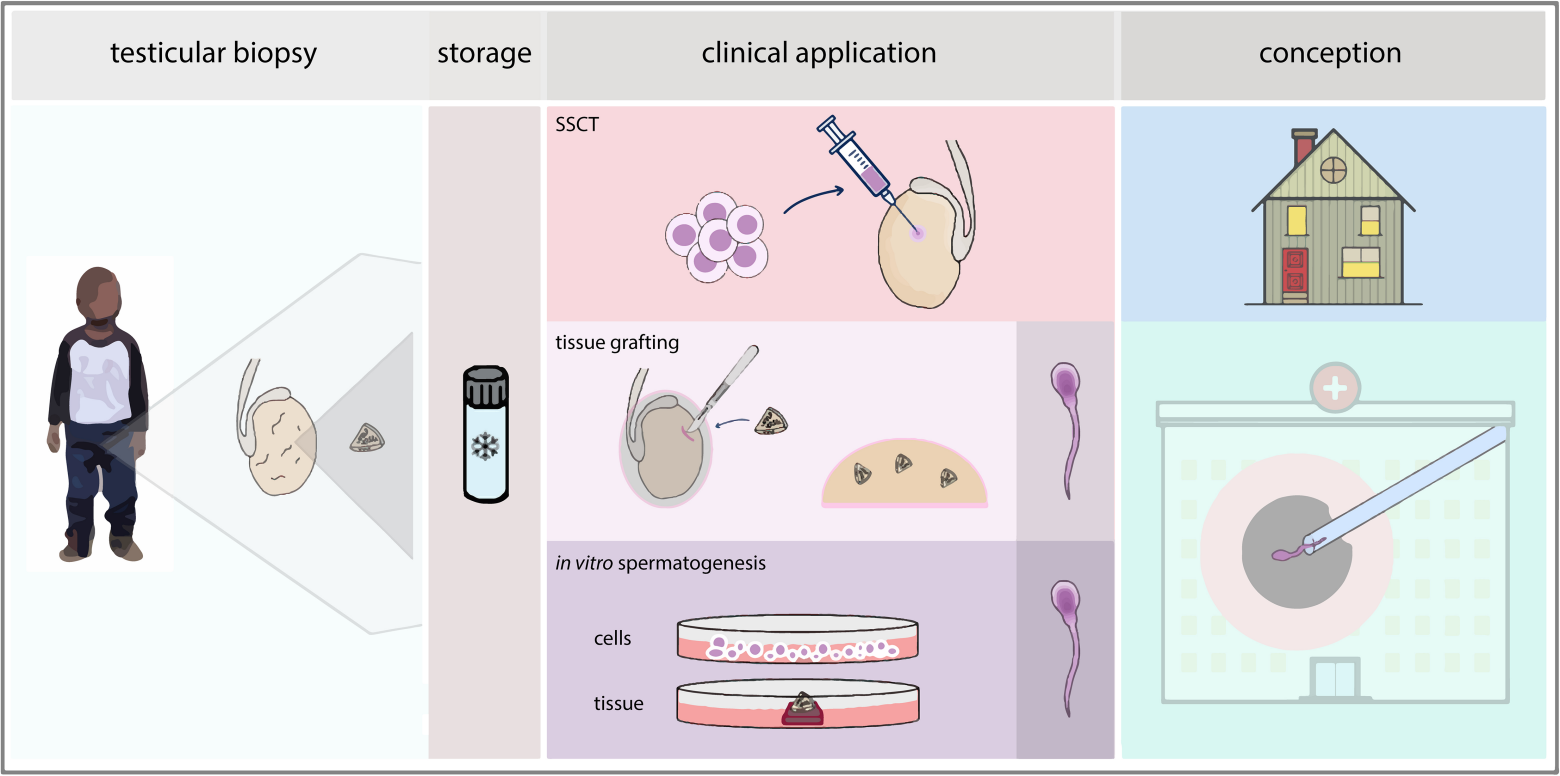
Pathways in fertility restoration. After cryopreservation of a testicular biopsy, clinical application of SSC-based techniques can be sought *via* SSCT, testicular tissue grafting or *in vitro* spermatogenesis, either through cell-based or tissue-based culture. For SSCT, natural conception could be achieved whereas for therapies based on grafting or *in vitro* spermatogenesis, an ICSI procedure would be performed using elongated spermatids derived from the technique.

In the male, functional spermatogenesis is crucial for fertility. Spermatogenesis is initiated at puberty and relies on functional SSCs, which are located at the basement membrane of the seminiferous tubules within the testes. The SSCs will either self-renew to maintain the number of stem cells or become differentiating spermatogonia that will develop into spermatocytes which, after two subsequent meiotic divisions, form haploid spermatids that will ultimately give rise to spermatozoa. Spermatogenesis is tightly regulated by the surrounding testicular somatic cells including Sertoli cells, peritubular cells and Leydig cells (2). Irradiation and the majority of chemotherapeutic agents use mechanisms that target proliferating cells which, unfortunately, include the proliferative SSCs, thereby impairing spermatogenesis (3). Prior to such a gonadotoxic treatment, adult men are given the option to cryopreserve sperm to enable them to sire a child *via* current fertility treatments. For young boys who have to undergo a gonadotoxic treatment, but do not produce sperm yet, there is currently no option to preserve and later on restore their fertility. However, the immature pre-pubertal testis does contain SSCs and cryopreservation of a testicular biopsy prior to the gonadotoxic treatment is offered in multiple medical centers around the world. Although still under development, SSC-based treatments thus provide an opportunity to preserve and restore fertility for pre-pubertal boys (4, 5). The development of methods for preservation and restoration of fertility has become an important aspect of research to further enhance the quality of life for male patients requiring gonadotoxic treatment during childhood. In trying to offer fertility preservation and restoration options, many avenues have been pursued through use of the SSCs residing within testicular tissues after birth (**Figure 1**). With the *in vivo* capacity of developing into sperm, SSCs are prime candidates for possible interventions to restore fertility, which include spermatogonial stem cell transplantation (SSCT), testicular tissue grafting and *in vitro* or *ex vivo* spermatogenesis.

In this review we will pinpoint which hurdles still need to be overcome in (preclinical) research to fulfill the promise of SSC-based approaches, in anticipation of providing these fertility treatments for patients.

## Tissue Sources for Research

The use of testicular tissue is a prerequisite for preclinical research on SSC-based techniques. In designing research and moving towards clinical applications, the target patient group should always be taken into consideration and the tissues used should represent this population as closely as possible.

In addition to a scarcity of available human testicular tissue for research in general, not all testicular tissues are equally suited for all lines of research in SSC-based approaches, as inherent differences can affect the presence and functionality of specific cell populations present within the testicular tissue that might influence the success rate of SSC-based research (**Table 1**). This is illustrated by the fact that prenatal, neonatal and adult tissues show distinct germ cell populations (6) and even within prenatal tissues differences in spermatogonial populations are evident between the sequential trimesters (7, 8). In addition, testicular somatic cells gain maturity over time, thereby altering the testicular micro-environment in the tissues used for research.

**Table 1:** Suitability of various testicular tissues as sources for preclinical research on fertility restoration techniques.

Human tissue	SSCT	Grafting	*In vitro* spermatogenesis
Fetal tissue, no genetic abnormalities relating to reproduction	+-	+	+
Neonatal tissue (of child up to 1 month of age), no genetic abnormalities relating to reproduction	+-	+	+
Pre-pubertal tissue, no genetic abnormalities relating to reproduction	+	+	+
Adult tissue, normospermia (e.g. retrieved from orchiectomy in treatment of prostate cancer)	+	-	-
Adult tissue, influenced by hormonal treatment prior to sex reassignment surgery	+	+*	+*
Adult tissue, obstructive azoospermia	+	-	-
Adult tissue, non-obstructive azoospermia: maturation arrest	+-	-	-
Adult tissue, non-obstructive azoospermia: SCO-syndrome	-	-	-
Adult tissue, Klinefelter syndrome or likewise genetic abnormalities	-	-	-
Adult tissue, testicular cancer	-	-	-
Adult tissue, epididymal disorders	+	-	-

**+** appropriate source for preclinical research; **+-** use with caution; **-** advised not to use for preclinical research; *suitable when spermatogenesis is reduced to spermatogonia only. To distinguish between the different SSC- based techniques, shading correlates to the colors in the figure.

For preclinical research on SSC-based therapies there is a clear preference for the use of pre-pubertal tissue, both because of good representation of the intended patient population as well as the technical suitability of this tissue for these studies.

When pre-pubertal material is not available, research on SSCT can use adult testicular tissues, preferably of those patients in which the functionality of the SSCs has been established through histological detection of complete spermatogenesis. Tissues bearing pathologies that affect the spermatogonial population, such as congenital abnormalities or acquired Sertoli cell only (SCO) syndrome, are unsuitable for *in vitro* studies on SSC proliferation because of the absence of (functional) SSCs. Pathologies which only affect germ cells during their post-spermatogonial differentiating stages, as is the case in non-obstructive azoospermia patients with maturation arrest at the level of meiosis or spermiogenesis, may leave spermatogonial function unaffected. However, the level of maturation arrest may influence culture outcomes when propagating SSCs, as *in vitro* culture of testicular cells with early (pre-meiotic) maturation arrest may lead to more colonies, with larger diameters, than cultures using testicular cells of tissues with late (post-meiotic) maturation arrest (9).

For research on testicular tissue grafting and *in vitro* spermatogenesis, adult testicular material is unsuitable as study results would be influenced by either the presence of already ongoing spermatogenesis or, in its absence, by factors such as maturation arrest hampering any possibility of differentiation. The use of mouse neonatal or pre-pubertal testicular tissue for grafting in mice has been successful in the derivation of fertilization-capable sperm from the graft (10). However, caution is warranted with optimization of grafting protocols using human neonatal or fetal tissues, as the immature population of germ and somatic cells may respond differently than the germ cells present within the tissue of the pre-pubertal patient population.

Generally, testicular tissues derived from patients with underlying conditions, or undergoing treatments that affect meiosis and/or spermiogenesis, such as hormonal treatments for gender dysphoria (11, 12) or infectious diseases (13), could be used under the assumption that spermatogonial function is not affected. It is known that the vast majority of testicular tissue obtained during orchiectomy in transwomen contains spermatogonia (14). However, to our knowledge, the effects of these conditions on SSCs have not been systematically studied.

Likewise, when a patient has already started chemotherapy or radiation regiments, the SSCs within their testicular tissue may have deteriorated or undergone changes that will cause impairment or apoptosis of SSCs *in vitro* (15–17).

Finally, the use of tissues containing testicular cancer is not recommended for any preclinical SSC-based research as cellular function may be influenced by the cancerous cells. In the case of germ cell neoplasia *in situ* (GCNIS) these cancerous cells, which originated from precursors of SSCs, will have many molecular characteristics similar to SSCs, which may influence study outcomes (18).

To interpret results in preclinical research on SSC-based approaches, thorough documentation of patient characteristics, demography and (testicular) pathology of tissues used in these studies is essential.

## Cryopreservation

For the future clinical application of SSC-based therapies, the testicular tissue biopsies are cryopreserved until the patient has a wish to have children, years after the pre-pubertal tissue biopsies were taken. During the freezing process of this testicular tissue, testicular cells might suffer from cryogenic damage caused by the formation of ice crystals. In an isotonic solution, water will crystallize spontaneously at ‐10°C (19). As a result, the osmolarity of the solution will increase, which will cause the cells in solution to lose intracellular water. When the cooling rate is too high, the cells are not able to lose all the intracellular water in time and intracellular ice crystals will be formed. When the cooling rate is too low, the cells will be exposed to a hyper‐osmotic environment for too long, which will cause cell shrinking and apoptosis. To prevent these forms of cryodamage, different freezing methods, devices and cryoprotectants can be used for correct storage of biopsies, to allow future use of the tissue in SSC-based therapies (19).

The freezing of either a testicular cell suspension or testicular tissue have been proposed as options to cryopreserve SSCs. The freezing of testicular tissue has preference due to greater viability of germ cells and in particular SSCs, compared to the freezing of a testicular cell suspension (20). Furthermore, the cryopreservation of intact testicular tissue fragments permits the future opportunity to pursue tissue-based therapies as well as retaining the option to generate a cell suspension from the testicular fragments for stem cell-based therapies. For cryopreservation, the testicular tissue is divided into small fragments (approx. 3 mm^3^), which are deposited in cryotubes or cryostraws containing a cryoprotective medium.

Currently, no standardized cryopreservation method has been established that is optimal for male fertility preservation. However, the most commonly used freezing protocol is controlled slow freezing (CSF) with seeding. In this method, testicular fragments in 5% dimethylsulfoxide (DMSO) are cooled with a rate of 1°C/min, maintained at 0°C for 5 min, followed by further cooling at 0.5°C/min until −8°C for hand seeding and continued freezing until −40°C and subsequently, after a short stop, to −70°C with a cooling rate of 7°C/min before transfer to liquid or vaporized nitrogen (21).

An alternative to slow freezing is the use of solid-surface vitrification (SSV). Using this method, a droplet of DMSO-based cryoprotectant containing tissue fragments is exposed to liquid nitrogen and subsequently stored (22). However, slow freezing seems to provide greater protection to testicular tissue than SSV. This is in line with other studies reporting the ability of a slow freezing protocol to maintain tubular architecture and integrity of pre-pubertal human testicular tissue fragments (21, 23–31).

Besides the different freezing regimes, various cryoprotectant freezing media can be used. To examine the effect of cryopreservation on testicular tissue fragments, Keros and colleagues analyzed morphology and structure of the tissue as well as the presence of MAGE-A4+ spermatogonia using light- and electron microscopy (21). In these experiments, the fragments were thawed and subsequently cultured for one day at 33°C in 5% CO_2_ prior to fixation. The results indicate that a freezing medium containing 5% DMSO (0.7 mol/l) during cryopreservation causes a reduced amount of cryodamage in comparison to other cryoprotectants such as 1.5 mol/l 1,2-propanediol (PrOH). Over 90% of MAGE-A4+ cells were undamaged (i.e. not detached from the basement membrane or neighboring Sertoli cells) when using DMSO, compared to 60% using PrOH.

This is in agreement with results showing that cryopreservation of testicular tissue by slow-freezing in 5% DMSO does not seem to affect the long-term culture of human testicular cells with regard to the presence of germ cell and somatic cell populations (32). Further optimization and possible addition of novel cryoprotectants, such as the antioxidant pentoxifylline (33) to the freezing medium may further improve the preservation of human SSC viability. Our research group cryopreserved testicular tissues obtained from orchiectomies of two adult patients with prostate cancer using various protocols in a pilot study. We compared cryoprotectant TEST-yolk buffer (TYB) with 7.5% human serum albumin (HSA, Albuman), 5% DMSO with 5% HSA and 8% DMSO with 20% HSA using a controlled slow freezing method described for tissue CSF as described above (21). Furthermore, we tested other freezing methods by bringing the straws with tissue fragments and cryoprotectant directly in the nitrogen vapor or using a shorter method of controlled slow freezing originally designed for cryopreserving sperm (CSFS), during which cryostraws were cooled with 0.5°C/min to 5°C followed by a cooling rate of 2°C/min until the samples reached 2°C. Finally, the samples were cooled until ‐80°C with a cooling rate of 10°C/min. No seeding was performed in this method. Morphology of the tissue was assessed after a 24-hour culture compared to fresh tissue by MAGE-A4 staining. Long-term cultures of isolated testicular cells from these tissues were assessed as well, including by SSC colony counts. Morphological damage was most pronounced in all CSFS frozen tissue and was also found to be increased in all those testis fragments cryopreserved in TYB-containing medium compared to align with DMSO-containing medium. The immunostaining of MAGE-A4+ cells in these tissues, 24 hours after culture, reflected these results. When testicular cells were isolated and cultured, cells frozen by CSFS and in nitrogen vapor of one patient did not survive long term culture. However, the cultures of isolated cells from testicular fragment frozen tissue in all other conditions were all capable of SSC colony formation, indicating presence and proliferation of SSCs. Collectively, the slow freezing method CSF (21) with 5% or 8% DMSO as a cryoprotective agent appears to be favorable for cryopreserving testicular tissue for future *in vitro* spermatogonial proliferation prior to transplantation as a fertility restoration treatment.

The fertility restoring potential of cryopreserved tissue has often been investigated with regards to testicular grafting, with the use of both fresh (10, 34, 35) and cryopreserved tissues (36–40) which have been shown to be successful in pig, mice and primate models. Direct comparisons of the use of fresh versus frozen-thawed tissues intended for tissue grafting have been performed in various species, but show variable results. In mice, spermatogenic progress within the testicular fragments after grafting was similar between grafted fresh tissue fragments and fragments that were frozen-thawed prior to grafting (41). In a later study, a lower number of intact tubuli at one day after grafting within grafted frozen-thawed tissues was found, but at later timepoints, up until two months, no differences were observed between grafted fresh and frozen-thawed tissues (42). Fayomi et al. did not show any differences between fresh and frozen-thawed conditions regarding graft weight, stage of spermatogenesis or percentage of tubuli with complete spermatogenesis, whether the grafts were ectopically or orthotopically placed (43). The use of human frozen-thawed testicular tissues xenografted in mice resulted in similar levels of structural integrity and germ cell survival as the use of fresh tissues, although the number of Sertoli cell-only (SCO) tubules was higher in the frozen-thawed tissue group (44).

For *in vitro* spermatogenesis with testicular tissue, cryopreserved testicular tissues have also been tested compared to fresh testicular organ culture. The organ culture used was first described for mouse testis fragments (45). This group compared fresh with cryopreserved mouse testis tissue with regard to their ability to develop spermatogonia to germ cells of late meiosis onwards under the reporter of *Gsg-*GFP (46). They applied uncontrolled slow freezing, using a Bicell biofreezing vessel, with a cooling rate of 1°C/min until -80°C before plunging in liquid nitrogen, using either 1.5 mol/l (11%) DMSO, 1.5 mol/l PrOH or Cell Banker 1 as cryoprotectant, or vitrification with Stem Cell Keep as cryoprotectant. They found that slow freezing with DMSO and vitrification with Stem Cell Keep showed similar progression of spermatogenesis as fresh cultured tissue, based on GFP expression levels. Also, for human testicular organ culture the use of fresh and cryopreserved tissues using CSF with 5% DMSO were compared (47). Reassuringly, no significant difference in culture outcome on spermatogonial maintenance and proliferation and hormone production between fresh and cryopreserved testicular fragments of human pre-pubertal boys could be found (47).

## SSC-Based Techniques

### *In Vitro* Proliferation and Transplantation of SSCs

Auto-transplantation of SSCs harvested from the patient prior to treatment could provide recolonization of the seminiferous tubules and subsequent *in vivo* spermatogenesis, in theory restoring fertility and the ability of the patient to achieve progeny through natural conception (**Figure 1**). To reintroduce the SSCs into the human testis, the preferential method seems the ultrasonically-guided injection of cells in the rete testis, a comprehensive review of which is written by Gul et al. (48).

Brinster et al. were the first to demonstrate the process of SSCT to be successful in establishing colonization and spermatogenesis in recipient testes in mice (49, 50), as well as in resulting in offspring with the donor haplotype (50). Since then, auto- or allo-transplantation of SSCs has been achieved in various mammals, including rodent and non-rodent species, as reviewed by Takashima & Shinohara (51), and non-human primates (52, 53), resulting in functional spermatogenesis within the recipient animals.

In humans, clinical trials with SSCT have not yet been established; a single report on SSCT in seven men receiving injections of cryopreserved testicular cells has not been followed up by a report on the outcome of these procedures (54). However, from studies in mice it has become clear that transplanted numbers of SSC colonies gradually decrease during the homing process after transplantation (55) and that the concentration of transplanted SSCs is highly linked to the success of colonization of SSCs (56) and donor-derived spermatogenesis within the recipient testis (57).

Because only biopsies of limited size can be collected from patients, propagation of SSCs retrieved from these biopsies will be necessary to achieve sufficient numbers for successful transplantation and recolonization of the seminiferous tubules within the recipient testis. Kanatsu-Shinohara et al. first demonstrated effective long-term propagation of murine SSCs, which were able to restore fertility in sterile recipients (58). In humans, long-term propagation of testicular cells was demonstrated to be possible as well, with cultured testicular cells from both adult (59) and pre-pubertal tissues (60). Identity and propagation between two time points in culture of these human SSCs could be demonstrated through xenotransplantation into sterile mice, by counting donor SSCs that had migrated to the spermatogonial stem cell niche within the seminiferous tubules.

Still, the identification of SSCs within a testicular tissue or cell culture *in vitro* is a major challenge. Testicular cell cultures include both somatic cells and germ cells, the latter of which is a heterogeneous group of spermatogonia of varying states of differentiation. At this moment there is no agreement on a specific, unambiguous marker for human and/or non-human primate SSCs *in vivo* and *in vitro*. For many currently used markers, including ITGA6, KIT, GPR125 and DAZL, expression is not strictly limited to spermatogonia and can also be found in testicular somatic cells (61, 62). For other markers, spermatogonial expression of the marker is controversial, as not all study results are in agreement. This is the case for GFRA1, THY1 and UCHL1 (22, 61, 63–79). Expression of certain markers can also rely on the developmental stage of the testicular tissue; for instance, expression of POU5F1 is limited to subpopulations of fetal and neonatal germ cells (gonocytes) in humans and expression is downregulated along with progressing differentiation (7, 8, 18, 80).

Single-cell sequencing studies have identified various ‘states’ of germ cells, with progression of development of differentiation from State 0 to State 4, where each state is characterized by a unique set of markers, although at times partially overlapping (6, 81). UTF1 and PIWIL4 are examples of markers expressed in the earliest state, with PIWIL4 being the more specific marker (6, 82, 83). Another newly identified candidate marker for undifferentiated spermatogonia, carrying SSC characteristics, involves the LPPR3 protein (6).

Furthermore, it is uncertain whether various testicular cell types retain their transcriptomic and metabolic signatures when isolated from their natural niche (62). Due to these dynamic processes, the heterogeneity of the testicular cell populations and the many potential markers to choose from, it is difficult to establish inter-study comparisons of culture outcomes and efficiency with regard to SSC propagation. To extend studies into the field of SSCs and their potential use, the scientific community would benefit from more insight into specific markers for clearly-identified spermatogonial populations, combined with functional studies as to their SSC potential with regard to *in vitro* use. In identifying markers, a focus on surface markers as opposed to nuclear markers may be more beneficial for isolation and enrichment of SSCs from cryopreserved biopsies or after culture.

Currently, the gold standard to demonstrate SSC functionality is still the (xeno)transplantation assay, in which donor-derived SSCs are shown to be capable to migrate to the stem cell niche within the seminiferous tubules of the recipient testis and, in a compatible species, initiate and maintain donor-derived spermatogenesis, although this latter part cannot be demonstrated in human to mice xenotransplantation assays.

In working towards clinical application of SSCT, study protocols have to be adapted to clinical requirements and regulations. Therefore, it will be essential to work towards xenofree, clinical grade media and methods in compliance with good manufacturing practice (GMP) and good clinical practice (GCP) systems, while adhering to the regulations regarding the production of advanced therapy medicinal products (ATMP, regulation no. 1394/2007 [European Medicines Agency (EMA)] (84).

The culture methods and media should support SSC proliferation while preventing their differentiation. Besides basic nutrients, additional components such as specific cytokines, metabolites, hormones and other signaling molecules, that are known to stimulate spermatogonial proliferation, may be included in the culture medium. In 2003, Kanatsu-Shinohara et al. developed a successful culture protocol, using medium based on StemPro-34 Serum Free medium, for long-term *in vitro* propagation of murine SSCs (58). Although a comparable medium can also support long-term propagation of human SSCs (59, 60), overgrowth of testicular somatic cells within the culture system remains problematic, as the SSC signature is diluted over time (62). The presence of feeder cells is beneficial to the survival of SSCs as they provide mechanical and metabolic support and paracrine signals (85, 86). The use of exogenous feeder layers however is unsuitable for clinical implementation of the technique. Therefore, somatic cells which support SSC proliferation and which are already present within the testicular suspension, isolated from a testicular biopsy, are used for SSC culture. To prevent overgrowth of these somatic cells, the culture method and medium used should steer towards an optimal balance between the growth of somatic cells and SSCs. Alternatively, the testicular somatic cells may be replaced by a (synthetic) matrix for cell support, combined with supplementation of the medium with nutrients and growth factors to ensure the metabolic support for SSCs otherwise provided by the feeder cells. This process however heavily relies on the ability to isolate and remove the somatic cell population from the germ cell population prior to culture, which is currently limited by the lack of a specific SSC marker (87, 88).

Optimization of the method for *in vitro* culture of human SSCs is thus a necessary step prior to clinical implementation. Investigation of the SSC niche in humans might be a pivotal step in identifying the factors necessary for effective maintenance and propagation of SSCs (89).

### Grafting of Testicular Tissue

*In vivo* spermatogenesis can also be achieved through autologous grafting of immature testicular tissue fragments underneath the skin of the patient (**Figure 1**). Through angiogenesis, the tissue receives endocrine signals from the body circulation which enable spermatogenesis within the tissue (90, 91). After full progression of spermatogenesis, spermatids can then be harvested from the retrieved graft and used in current assisted reproductive techniques like intracytoplasmic sperm injection (ICSI).

Testicular (xeno)grafting has been shown to lead to successful spermatogenesis in testicular tissues of many mammalian species, including mice, pigs and monkeys (34, 35, 37), subsequently leading in some cases to live offspring through application of ICSI (10, 35, 40, 43, 92). Host conditions may impede full spermatogenesis within xenotransplants with cryopreserved pre-pubertal tissues (39), although in non-human primates complete spermatogenesis has been achieved with autologous transplantations of fresh testicular pre-pubertal tissue as well as with xenografts of similar origin (34, 93).

In the xenografting of non-human primate testicular tissue to mice, the spermatogenic maturation depended on the location of the graft, with orthotopic (intra-testicular) grafts leading to further spermatogenic progression in a higher number of grafts than ectopic grafts under the skin of the back (38, 94). The ectopic location of the grafts in these experiments may have caused a higher tissue temperature and different hormone concentrations than those physiologically present in the scrotal area, potentially contributing to spermatogenic arrest. Recently, Fayomi et al. succeeded in fertilization of primate oocytes using autologous graft-derived primate spermatozoa, resulting in the birth of a healthy rhesus macaque (43). As of yet, no autologous grafting has been described with human tissue; only xenografts to mice have been studied. The use of human tissue in xenografts to mice has not yet resulted in complete spermatogenesis (95, 96), although meiotic activity could be observed (44).

For clinical use, the quantity of collected material is likely small, which could represent an obstacle for the success of this technique. Low survival rates of grafted tissues have been reported, potentially necessitating unilateral orchiectomy to retrieve sufficient amounts of material (39).

Success of this technique also relies on adequate vascularization of the grafted tissue, delivering both the necessary oxygen and hormones for SSC proliferation and differentiation. Hypoxia in the graft center may lead to loss of testicular tissue and all germ cells within (42). Vascularization of human grafts has been shown to benefit from a short-term culture supplemented with vascular endothelial growth factor (VEGF) prior to placement of the graft (93), but additional growth factors other than VEGF may be necessary for successful graft survival.

### *In Vitro* or *Ex Vivo* Spermatogenesis

Spermatogenesis is one of the most complicated developmental processes in the human body, and thus remains very challenging to re-create by *in vitro* differentiation of stem cells or *ex vivo* cultures of testicular fragments (**Figure 1**). Nevertheless, application of *in vitro* or *ex vivo* spermatogenesis holds great potential for future fertility restoration or preservation, as haploid spermatids derived from these methods could be used for assisted reproductive technologies such as ICSI.

The use of SSCs as basis for *in vitro* spermatogenesis (97–101), isolated from a testicular biopsy taken prior to gonadotoxic treatment, would have high clinical relevance. Although many efforts have been made to re-create spermatogenesis *in vitro* using pluripotent stem cells (PSCs) (102, 103), blastocyst embryos to isolate the required embryonic stem cells (ESCs) will likely not be available for clinical use. Moreover, even though induced pluripotent stem cells (iPSCs), generated by genetic reprogramming of cells from the patient’s own somatic tissues could be a possible alternative source, the safety of the use of iPSCs for reproductive purposes has still not been sufficiently investigated. However, regardless of PSCs or SSCs as the cell type of origin, the most challenging spermatogenic process to mimic *in vitro* is the process of meiosis, the process by which genetically different haploid spermatids are produced *via* two successive meiotic cell divisions. Although several studies reported generation of round spermatid-like cells *in vitro* using SSCs (97, 98, 100, 101), only a few studies (98, 101) investigated the key meiotic events that are required for successful meiosis and true *in vitro*-derived gametes (104). When mouse SSCs were induced to complete meiosis, using immortalized Sertoli cells as feeder layer in a three-step induced culture system, despite the *in vitro* formation of pachytene-like spermatocytes, the homologous chromosomes did not display full synapsis and no meiotic crossovers were formed. Despite these meiotic problems, many cells still proceeded to the first meiotic division (MI), occasionally even forming round spermatid-like cells (98). Apparently, these aberrant spermatocytes were not timely eliminated by the meiotic checkpoints that normally (*in vivo*) induce apoptosis in order to prevent possible generation of aneuploid sperm (105). Because of a lack of meiotic crossovers in the *in vitro-*generated pachytene spermatocytes, the MI-spermatocytes did not form chiasmata (physical connection between the homologous chromosomes) and thus displayed univalent (pairs of sister chromatids) instead of bivalent (pairs of homologous chromosomes) chromosome pairs, which will almost certainly cause subsequent formation of aneuploid spermatids. Meanwhile, Sun et al. reported complete chromosome synapsis and meiotic crossover formation *in vitro* using human male germline stem cells (101). However, the detection of chiasmata in MI-spermatocytes was not described.

The difficulty in mimicking spermatogenesis *in vitro* could be due to the lack of a suitable testicular micro‐environment needed to successfully support the spermatogenic process. Spermatogenesis *in vivo* requires spatio-temporal interactions between germ cells and testicular somatic cells. Therefore, including an *in vitro* somatic niche in culture could be instrumental in supporting the germ cells. In mice and humans several essential regulators, such as retinoic acid and gonadotropins, have been identified and are appropriately discussed in reviews such as that of Rombaut and colleagues (106).

Testicular somatic cells, including peritubular myoid cells, Leydig cells, Sertoli cells and endothelial cells, as well as immune cells, play important roles in supporting spermatogenesis. Unlike *in vitro* spermatogenesis, in *ex vivo* spermatogenesis methods where testicular fragments are cultured instead of isolated cells, testicular organization is still in place during culture, thereby circumventing the need to structurally re-create the right microenvironment.

The first successful *ex vivo* spermatogenesis was demonstrated in 2011 (45, 107). In this study, murine testicular fragments were cultured at a gas-liquid interphase using an agarose stand and *ex vivo* elongated spermatids were identified after five weeks of tissue culture and could be used for the generation of live offspring. Repeating this method for rat immature testicular tissue resulted in progression of spermatogenesis up to round spermatids (108). Studies using human testicular tissue for *ex vivo* spermatogenesis showed no initiation of meiosis (109, 110). Using histological analysis, one study reported that spermatids could be observed morphologically in *ex vivo* cultures with human testis tissue (111). No studies using human or non-human primate tissues describe the production of spermatids that were shown to be capable of fertilization.

Despite the achievements that have been reached with *ex vivo* spermatogenesis using tissue culture, the efficiency to perform *ex vivo* spermatogenesis is very low. More research is required to improve the efficiency and further translation of the method for primate testicular tissue.

More fundamental knowledge on non-human primate and human spermatogenesis and the differences with that of rodents will be helpful to successfully mimic this process through either *in vitro* or *ex vivo* spermatogenesis.

## Safety

### Biopsies for Fertility Preservation

As any medical procedure may involve short- or long-term health risks, it is important to consider the impact that taking testicular biopsies may have on the patient. In many cases, the patient’s parent(s) or caretaker(s) will have to make the decision for the child to undergo this procedure and they will have to be duly informed of its potential risks, including those of the general anesthesia under which the procedure is performed. These risks however may be limited by concomitant execution of other medical procedures that are inherent to the initial (cancer) treatment, such as central venous line placement or bone marrow aspiration. Worries about the risks of the biopsy itself comprise the chance of acute post-operative complications and chronic changes within the testicular tissue. Post-operative complications include post-operative bleeding and wound infection. Rates of occurrence of these complications are low and vary between 0 – 3.8% (112–115). Transiently, extra- and intra-testicular hematomas may occur (112). Additionally, when the testicular biopsy is performed, to reduce the burden of repeated anesthesia, as an additional procedure during general anesthesia for a concomitant procedure, such as central venous line placement or bone marrow aspiration, this may lead to increased post-operative pain levels compared to the main procedure only, potentially necessitating additional analgesics in these patients (116).

Unilateral testicular biopsies of a maximum of 1 ml or 50% of total testicular volume of pre-pubertal patients did not lead to decreased growth, compared to the contralateral non-biopsied testis, as measured by ultrasound during one year after surgery (112). In 6.3% of the patients fibrotic testicular lesions were found using ultrasound as part of a long-term follow up (112). Although gonadotrophin levels have been observed to be abnormal in the follow-up of pre- and peri-pubertal boys from whom a testicular biopsy was taken, the incidence of these abnormal levels matches those of childhood cancer survivors who have not undergone testicular biopsies (113). Similarly, results of semen analyses of patients who had undergone testicular biopsies prior to treatment (88% of which with alkylating agents) showed comparable rates of normo-, oligo- and azoospermia to large-scale cohort studies of childhood cancer survivors (113).

In conclusion, the reproductive health of patients undergoing testicular biopsies does not seem to decline as a result of the testis biopsy procedure.

For fertility preservation, the biopsies are subsequently stored in nitrogen. Although cryopreserved tissue function has been shown to be similar to that of fresh tissue, as described above, the effect of cryopreservation on the genetic and epigenetic status of cells within cryopreserved tissues is largely unknown. Despite the availability of studies on molecular changes on cryopreserved sperm, oocytes and embryos (117), studies researching the effect of cryopreservation on SSCs are limited. In mice, SSCT was as successful with freshly isolated germ cells as with germ cells isolated from long-term cryopreserved tissues, in establishing spermatogenesis in recipient animals and subsequent fertilization of murine oocytes through ICSI (118). The resulting offspring did not show a significant amount of DNA copy number changes (chromosomal deletions, duplications) nor changes in DNA methylation patterns in whole-genome DNA analysis of liver tissue, compared to the offspring of wild-type mice, obtained through natural conception, suggesting that potential DNA methylation changes of SSCs in cryopreserved tissues are reversed upon transplantation, or in the offspring.

### Safety of SSCT for Patients and Progeny

A major concern with regard to SSCT in cancer patients is the possible reintroduction of malignant cells from the original biopsy during the autotransplantation of (cultured) testicular cells to the patient who recovered from cancer. Research data indicate that acute lymphoblastic leukemia (ALL) cells do not survive for longer than 16 days in a human testicular cell culture system (119). *In vitro* propagation to increase SSC numbers prior to autotransplantation therefore seems to have the additional benefit of eliminating ALL-cells, although this may not be the case for other cancer types. Further purification methods, such as immunomagnetic bead-based sorting (MACS) using a combination of spermatogonial markers showed promising results in a mouse model (120). In contrast, the use of Percoll density gradients appeared insufficient to prevent transmission of leukemic cells to the recipient mice (120). Fluorescence-activated cell sorting (FACS) also showed promising results in experiments with human cells. The combined use of the markers EPCAM, HLA-ABC and CD49e allowed the separation of putative spermatogonia and leukemic cells prior to xenotransplantation. Tumor formation was only observed in the testis of transplanted animals having received the leukemic fraction, and tumors were absent in animals receiving the spermatogonia-enriched fraction, whilst the latter also showed the highest number of spermatogonial colonies (121). However, such separations are highly dependent on knowledge of specific antigens that are different between spermatogonial and cancerous cells. However, cancer cells are known to express many genes that are normally only present within germ cells (122, 123), including antigens that may be used for sorting (124). In addition, purification strategies need to be highly reliable, as only 20 leukemic cells are needed to cause a terminal relapse, as was shown in a rat model (125). Any patient-specific aberrations in tumor antigen expression could therefore be disastrous, when relying on the selection of cells by specific antigens. Prior to cell enrichment, a thorough characterization of the cancer cells from every individual patient would thus be pertinent.

Although a valid concern for cell culture and manipulation in general, the culture of human SSCs does not seem to induce chromosomal abnormalities (126, 127). This is similar to the genetic stability of long-term (over two years, with an approximate 10^85^-fold expansion) cultured murine SSCs, which have been shown to maintain their androgenic imprint based on five imprinted regions and their capacity for spermatogenesis after transplantation, leading to fertile progeny (128). DNA methylation patterns of cultured human testicular cells, sorted for ITGA6, do not overlap with those found in seminomas (127), indicating cultured cells do not acquire a seminoma signature. However, specific DNA demethylation patterns of paternally imprinted genes and concurrent DNA hypermethylation of maternally imprinted genes were observed in cultured and enriched human spermatogonia (126). It is currently unknown whether these changes are correlated with the fertilization potential of spermatozoa that would be derived from these spermatogonia, or whether they pose a health risk towards the patient or their offspring, although studies in mice do not seem to indicate this (129, 130).

As there has not yet been a clinical trial investigating the application of SSCT in human patients, no information regarding the long-term health in such patients is available. However, research in mice shows no difference in life-span up to the age of 18 months between transplanted and non-transplanted groups of busulfan-treated animals, with no increase in the occurrence of malignancies (129). Regarding the offspring, in non-human primates the sperm derived from the donor-SSCs after non-cultured SSC allogenic transplantation has been shown to possess competence for fertilization *via* ICSI and subsequent embryonic development up to the morula stage (52, 53). However, (epi)genetic stability of these embryos was not assessed and no progeny was derived in this study, limiting assessment of safety in respect to health of the procedure in primates. Nevertheless, in mice the long-term follow up of two generations of progeny of transplant-recipient mice showed no significant differences compared to the control group regarding congenital abnormalities, childhood development, lifespan, reproductive health and adult general health (130). These findings are supported by studies in multiple mouse strains reporting no chromosomal abnormalities or genetic deviations (131). To add, no changes were seen in DNA methylation patterns in spermatozoa derived from animals receiving SSCT of non-cryopreserved and uncultured spermatogonia, nor in spermatozoa and somatic cells of multiple organs of their first and second generation offspring (132). However, a later study on transplantation of freshly isolated spermatogonia did find that the expression pattern of histone lysines H4K5ac and H4K8ac in murine germ cells in the various stages of the seminiferous cycle in testicular tissue of animals having received SSCT differed from that in controls, as analyzed by immunohistochemistry. However, H3K4me3, H3K9ac, H4K12ac and H4K16ac of germ cells after SSCT were comparable to controls, as was their DNA methylation status (133).

### Safety of Graft Placement

As clinical trials have not commenced, no safety studies concerning human testicular grafts have been performed. While the main focus of studies has been to achieve spermatogenesis within the graft, little has been published regarding the (epi)genetic stability of the procedure and the cells within the graft, for both the recipient and his progeny. In mice, graft-derived spermatids showed normal fertilization capacity and progeny derived from graft-derived sperm showed good reproductive health, as demonstrated through mating experiments (10). However, general health of this offspring was not assessed.

Studies in testicular grafts of rhesus macaques showed good fertilization potential of graft-derived spermatids through testicular sperm extraction and intracytoplasmic sperm injection (TESE-ICSI), with good embryonic development up to blastocyst stage (34) and even a healthy live birth of one female (43). Behavior assessments of this graft-derived infant were age-appropriate and no health defects were reported by the authors (43).

Although these results are promising, the basis of the presumed safety of this procedure is still very small. Using immunohistochemistry markers on mouse tissue, it was shown that, compared to wildtype animals, testicular grafting does not alter the spermatogenic methylation patterns of germ cells in various stages of the seminiferous cycle as analyzed by immunohistochemistry analysis of expression of DNA methyltransferases -1 and -3A and 5-methylcytosine (DNMT1, DNMT3A and 5-MC) (133). Concerning histone modifications on H3K4me3, H3K9ac, H4K5ac, H4K8ac, H4K12ac and H4K16ac, only H4K5ac showed a significant difference in expression pattern between grafted and control groups (133).

To our knowledge, there are currently no other studies on the genetic integrity of spermatids derived from (xeno)grafts. Similarly, reports on assessment of the health of the recipient and their progeny are limited. Furthermore, in anticipation of the clinical use of this technique, it will be essential to ascertain whether the placement of an autologous graft from a cancer patient with potential infiltration of systemic cancer in the testis will pose a risk of recurrence of the cancer after grafting. Further studies are needed to establish reliable methods to secure transplantations of cancer-free materials only (5).

### Safety of *In Vitro* or *Ex Vivo* Spermatogenesis

#### Aneuploidy and Genomic Instability

For *in vitro* or *ex vivo* spermatogenesis, one of the foremost risks is the derivation of sperm with an incorrect number of chromosomes, referred to as aneuploidy. An estimated 20–40% of all human conceptions contains aneuploid cells, of which most are due to errors in meiosis (134). In all males, a proportion of sperm can be observed to be aneuploid (135, 136). Approximately 3–5% of spermatozoa of males with proven fertility are aneuploid, and these levels are significantly higher in infertile men (136–139). Aneuploidy in gametes may cause early pregnancy loss or severe developmental defects (140, 141). Meiotic problems, like incomplete chromosome synapsis and impaired recombination, are considered to be main factors in the emergence of chromosome nondisjunction and subsequent aneuploidy (138, 142, 143). In order to prevent generation of aneuploid sperm, key meiotic events such as chromosome synapsis, recombination and subsequent meiotic crossover formation, are strictly monitored by checkpoint mechanisms to timely arrest meiotic progression (105, 144). However, when spermatogenesis was re-created *in vitro* using mouse SSCs, these checkpoint mechanisms did not appear to be functional (98). For *ex vivo* cultures of testicular fragments, most meiotic events or meiotic checkpoint function have not yet been systemically investigated. Since meiotic DNA damage repair, recombination and checkpoints appear not to be fully functional in current *in vitro* spermatogenesis protocols, these processes should be thoroughly investigated and monitored before considering clinical application of *in vitro* or *ex vivo* spermatogenesis.

#### Epigenetics

Epigenetic modifications, being DNA methylation, histone modification and the production of small non-coding RNAs, regulate many processes of spermatogenesis. Besides spermiogenesis, also spermatogonial differentiation steps and early meiosis are subject to epigenetic regulation. For example, some changes in DNA methylation may occur in spermatogonia and early spermatocytes (145). These processes include meiotic silencing of unsynapsed chromosomes (MSUC), XY-body formation and the histone-to-protamine transition during spermiogenesis (146). These are all crucial for germ cell function and post-fertilization embryonic development (147). The establishment of epigenetic patterns during human germ cell development is dynamic and age and phase-dependent (148, 149). Aberrant DNA methylation during spermatogenesis may impair spermatogenesis, causing infertility (150). Despite post-fertilization (embryonic) epigenetic reprogramming as a mean to prevent aberrant progeny, successful fertilization of oocytes with epigenetically abnormal sperm may still pose a risk, because some abnormal epigenetic marks may persist (151) and potentially influence embryonic development and health of the offspring. In couples experiencing recurrent pregnancy loss, sperm showed more DNA methylation abnormalities of multiple imprinted genes, compared to couples without history of miscarriage or infertility (152), illustrating the potential detrimental effect of epigenetic aberrations in germ cell function.

Moreover, long-term culture of primary cells in general may induce changes in DNA methylation (153). Therefore, when spermatogenesis is re-created *in vitro*, the epigenetic status has often been investigated to make sure that no aberrant epigenetic patterns are present in the *in vitro*-derived germ cells (100, 101).

When murine SSCs were used as a starting point to generate a multipotent adult GSC line (maGSCs) that could subsequently be induced to differentiate into haploid cells, these haploid cells showed incomplete epigenetic imprinting of the H19 gene (100). Nevertheless, a study using human male germline stem cells reported normal epigenetic status in round spermatids-like cells (101).

The research on epigenetic status of cells derived from testicular tissue cultures is limited. The short-term culture of rat fetal testicular tissue shows a similar chronology in epigenetic remodeling of DNA methylation patterns in gonocytes, compared to gonocytes *in vivo* (154). Similarly, Yokonishi et al. described a normal state of methylation in the offspring derived from the culture of cryopreserved mouse testicular tissue fragments (46). However, DNA methylation is not the only type of epigenetic regulation and more studies are needed to ascertain the epigenetic properties of cultured SSCs, spermatocytes and spermatids.

#### Limitations and Potential Risks of ROSI

Unlike *ex vivo* spermatogenesis, in which formation of elongated spermatids could be achieved that could be used for ICSI (45, 46, 155), differentiation of human SSCs by cell culture has so far, to our knowledge, only produced round spermatid-like cells (97, 98, 100, 101). Round spermatid injection (ROSI) technique would enable the use of these immature haploid precursors of spermatozoa to fertilize oocytes and give rise to the offspring. However, some concerns regarding ROSI have arisen as round spermatids and elongated spermatids have significant differences in chromatin structure (156). The use of round spermatids may not establish proper imprints or global methylation, thereby affecting embryonic development (157). Although in two human studies the children born after ROSI appeared healthy without any epigenetic problems (158, 159), data from a mouse study described that, in contrast of the normal paternal genome derived from mature spermatozoa, embryos derived from round spermatids appeared to have genome-wide aberrant DNA methylation of their paternal genome (160). This likely explains the observed poor development of these embryos. In addition, the overall low success rates of ROSI (161) compared with TESE-ICSI with testicular elongated spermatids may further limit the application of ROSI for human fertility restoration. Therefore, further development of these round spermatids or spermatid-like cells into more mature elongated spermatids is crucial for future clinical application.

## Concluding Remarks

As the patient population of pre-pubertal boys who have been offered fertility preservation slowly reaches their reproductive age, the issue of offering fertility restoration becomes more urgent. The research on SSCT, tissue grafting and *in vitro* spermatogenesis has concomitantly matured over the years and currently applications are on the horizon. For SSCT, the proof of principle has been delivered through allotransplantations in monkeys and the safety of the procedure has been extensively studied in mice. Potential for further optimization lays in *in vitro* human SSC propagation to increase their numbers and to purge cell suspensions of cancerous cells prior to transplantation. The use of testicular tissue grafting is also within reach of the clinic, with the existence of an autologous non-human primate model having resulted in healthy offspring. Challenges for grafting still exist with regard to the harvest of sufficient amounts of testicular tissue for grafting, the prevention of apoptosis within these grafts and assessment of the genomic status of the graft derived spermatids, as well as finding ways of preventing reintroduction of cancerous cells to the patient from within the grafted tissues.

Although spermatogenesis *in vitro* or *ex vivo*, either through cell or tissue culture, shows great promise with regard to the controlled production of spermatids, complete spermatogenesis with human tissue or isolated SSCs has not yet been indisputably achieved. Along with major concerns surrounding the genomic stability of haploid spermatid (like) cells generated by *in vitro* spermatogenesis, this technique does not yet seem ready for clinical application.

Despite these challenges, as continuous achievements are made within this field of research, all three avenues towards fertility restoration remain worth pursuing. To ensure progress, it is necessary to explore all options for tissue resources in preclinical research, although we should remain vigilant of the effect the tissue of choice has on study outcomes. In addition, more fundamental research towards identification of reliable *in vivo* and *in vitro* markers for identification and confirmation of testicular cell populations is vital for progress in this field.

For current and future patients, we should seek to offer comprehensible up-to-date and honest information on the progress of potential fertility preservation and restoration strategies and to inquire as to their wishes regarding these procedures.

## Author Contributions

IS and JM took the lead in drafting this manuscript, all other co-authors (JE, QL, AM, AAM, GH, AP, and CM) wrote sections of this manuscript. AAM, AM, AP, and JE contributed substantially to the cryopreservation section, while QL and GH contributed substantially in the (safety of) *in vitro* or *ex vivo* spermatogenesis. JM designed the figure and table. All authors took part in critical review and revising of the manuscript and approved the final version.

## Funding

This research was funded by ZonMW TAS, grant number 116003002. QL is the recipient of a China Scholarship Council grant (201706300107).

## Conflict of Interest

The authors declare that the research was conducted in the absence of any commercial or financial relationships that could be construed as a potential conflict of interest.

## Publisher’s Note

All claims expressed in this article are solely those of the authors and do not necessarily represent those of their affiliated organizations, or those of the publisher, the editors and the reviewers. Any product that may be evaluated in this article, or claim that may be made by its manufacturer, is not guaranteed or endorsed by the publisher.
